# Defining and Characterizing Postprandial Reactive Hypoglycemia

**DOI:** 10.3390/nu18050822

**Published:** 2026-03-03

**Authors:** S. Katherine Sweatt, Diana M. Thomas, G. Jake LaPorte, Skyler Chauff, Darko Stefanovski, Barbara A. Gower

**Affiliations:** 1 Department of Nutrition Sciences, University of Alabama at Birmingham, Birmingham, AL 35205, USA; bgower@uab.edu; 2 Department of Mathematical Sciences, United States Military Academy, West Point, NY 10996, USA; diana.thomas@westpoint.edu (D.M.T.); grover.laporte@westpoint.edu (G.J.L.); skyler.chauff@westpoint.edu (S.C.); 3 School of Veterinary Medicine, University of Pennsylvania, Kennett Square, PA 19348, USA; sdarko@vet.upenn.edu

**Keywords:** reactive hypoglycemia, insulin sensitivity, hunger, obesity

## Abstract

*Objective:* Individuals with reactive hypoglycemia (RH) may be more likely to develop obesity and type 2 diabetes, but the ability to identify RH has been hampered by the lack of clear criteria. This study used calculus-based curve parameters from a mixed macronutrient liquid meal test (MMTT) to define RH in men and women with obesity. *Methods:* A total of 69 non-diabetic adults aged 35 ± 8.3 years with obesity (BMI 32.3 ± 4.2 kg/m^2^) underwent a 4 h MMTT to define RH, and an intravenous glucose tolerance test (IVGTT) to characterize RH (via insulin sensitivity, the acute insulin response to glucose (AIRg), insulin clearance, and the disposition index). Perceived hunger and fullness were assessed by visual analog scale. *Results:* RH was defined using curve properties of the MMTT. A total of 19 of the 69 participants had a reactive hypoglycemic response to the MMTT. Glucose AUC and nadir were lower, timing of glucose nadir was earlier, and insulin sensitivity was higher in RH compared to non-RH. Sex (female) and race (AA) were significant predictors of RH presence. *Conclusions:* Among individuals with obesity, RH is characterized by greater sensitivity to insulin and greater disposition index. We introduce a novel and reproducible method to define RH using curve-based criteria from a mixed meal test integrated with gold-standard IVGTT-derived outcomes.

## 1. Introduction

Postprandial reactive hypoglycemia (RH) is defined as a rapid decline in blood glucose occurring 2–5 h after food intake, often followed by counter-regulatory mechanisms that stimulate hepatic glucose production [1]. RH is frequently observed in individuals with insulin resistance, hyperinsulinemia, or prediabetes, where insulin secretion is disproportionately high relative to carbohydrate intake [2,3]. In such cases, RH may serve as a marker of impaired glucose regulation and heightened risk for developing type 2 diabetes (T2D) [4,5]. However, RH remains poorly characterized, particularly in individuals with obesity, and there is limited understanding of how different testing methods, such as the mixed meal tolerance test (MMTT) and the intravenous glucose tolerance test (IVGTT), capture its underlying metabolic features. This gap in knowledge hinders the ability to define RH clinically and to determine its relevance as an early predictor of metabolic disease.

Glucose homeostasis is maintained by complex regulatory mechanisms. Following food intake, insulin is secreted to promote glucose uptake by insulin-sensitive tissues. When RH occurs, glucagon rapidly stimulates glycogenolysis and gluconeogenesis to rapidly rise glucose levels. If glucagon is insufficient, epinephrine is secreted from the adrenal medulla within minutes to further stimulate a rise in glucose and reduce glucose uptake in muscle and adipose tissue. Cortisol and growth hormone contribute over a longer time frame by limiting glucose disposal and enhancing hepatic glucose output [6]. Thus, RH elicits a cascade of endocrine and neuroendocrine counter-regulatory responses designed to oppose excessive postprandial glucose decline. However, elevated postprandial glucose production and impaired suppression of lipolysis in the context of RH may accelerate progression to T2D in individuals with insulin resistance, hyperinsulinemia, or prediabetes. Additionally, the rapid drop in glucose may increase hunger, potentially driving excess energy intake. Despite these implications, the metabolic and behavioral consequences of RH in individuals with obesity remain poorly defined. Determining how RH contributes to dysregulated glucose metabolism, energy intake, and long-term diabetes risk is critical to inform dietary and clinical strategies for prevention.

Initial hypotheses regarding the etiology of RH centered on abnormalities in insulin secretion, such as exaggerated postprandial insulin release [7,8]. Early studies reported that exaggerated insulin response to an oral glucose tolerance test could explain RH in both nonobese and obese individuals with impaired glucose tolerance; however, this was not consistent across all participants [7]. For instance, Hall et al. reported that only 16 of 40 individuals with RH demonstrated exaggerated insulin response, and insulin resistance values were not significantly different from the control group. Furthermore, 25% of RH participants demonstrated symptoms of hypoglycemia without a significant decline in glucose [2]. These findings underscore the heterogeneity of RH and highlight the need to better define its physiological characteristics and underlying mechanisms.

While RH may signal metabolic dysregulation, there are many limitations in studying RH. First, there is no consensus definition of RH and there is a lack of gold standard diagnostic criteria. Specifically, there are variations in glucose levels indicating hypoglycemia [9]. Thresholds such as <70 mg/dL are commonly used in practice, although more stringent cutoff values (e.g., <54 mg/dL) are considered for defining clinically significant episodes [6,10]. There are reports, however, that symptoms can occur at glucose levels above 70 mg/dL [2]. Therefore, other measures, such as the rate of glucose decline, may be more indicative of RH than glucose concentration. Data using mixed meals are limited, but mixed meals allow for investigation of glucose and insulin dynamics following a more realistic challenge than an oral glucose tolerance test. Additionally, there is no predictive model identifying RH. Characterizing the postprandial glucose and insulin responses that produce RH will provide more information to more accurately identify RH and determine health implications and treatments for individuals with RH.

The objectives of this study were to (1) define RH using the glucose and insulin responses to an MMTT and calculus-based curve parameters, (2) characterize RH using intravenous glucose tolerance test (IVGTT) with model-derived outcomes [insulin sensitivity (S_I_), AIRg, insulin clearance, and disposition index (DI)]; and (3) evaluate the postprandial hunger and satiety responses in healthy adults with obesity. Our a priori hypothesis was that individuals with RH in this cohort will have increased insulin sensitivity, disposition index, and AIRg; lower clearance; and earlier return to hunger.

This study is novel in several respects: (1) we define RH using novel mathematical variables in conjunction with a physiologically relevant MMTT, and (2) we characterize RH using an independent measure, the IVGTT. Finally, we build a predictive model for RH that may enable future screening and phenotyping in larger populations. This study addresses critical gaps in our understanding of RH, including its inconsistent physiological definition, limited characterization in individuals with obesity, and uncertain links to glucose metabolism, energy intake, and type 2 diabetes risk, and therefore lays the groundwork for dietary and clinical strategies to mitigate its impact.

## 2. Materials and Methods

### 2.1. Participants

Participants were healthy, African American (AA) and Caucasian American (CA) males and females aged 21–50 years with obesity. Race was determined by self-report. Exclusion criteria were type 1 or type 2 diabetes, polycystic ovary disease, weight change > 5 pounds in last 6 months, regular exercise >2 h per week, pregnancy, current breastfeeding, cholesterol medications, any diagnosed disorders of glucose or lipid metabolism, use of medication that could affect body composition or glucose metabolism (including oral contraceptives and blood pressure medications), current use of tobacco, use of illegal drugs in last 6 months, major food allergies or food dislikes, women with inconsistent or absent monthly menstrual cycles, and a medical history that counter-indicated inclusion in the study. Participants were evaluated for glucose tolerance using a 2 h oral glucose tolerance test, and only those who had 2 h glucose in the normal or mildly impaired range (≤155 mg/dL) were eligible for the study. The 155 mg/dL threshold was selected to exclude individuals meeting diagnostic criteria for impaired glucose tolerance (≥140 mg/dL) while allowing inclusion of individuals with modest post-load glycemic excursions common in obesity. This pragmatic cutoff enabled characterization of postprandial glucose dynamics within a metabolically at-risk but non-diabetic population without including individuals with overt dysglycemia. Participants were informed of the experimental design, and oral and written consent were obtained. The study was approved by the Institutional Review Board for Human Use at the University of Alabama at Birmingham (UAB).

### 2.2. Hormone Analysis

Concentrations of glucose, insulin, and C-peptide were analyzed in the Core Laboratory of the Diabetes Research Center (DRC). Glucose was measured in 3 µL sera using a glucose oxidase method (Stanbio Sirrus analyzer; Stanbio Laboratory, Boerne, TX, USA). This analysis had a mean intra-assay coefficient of variation (CV) of 1.21%, and a mean inter-assay CV of 3.065%. Insulin was assayed in 50 µL aliquots with immunofluorescence (TOSOH AIA-II analyzer, TOSOH Corporation, South San Francisco, CA, USA; mean inter-assay CV 4.42%; mean intra-assay CV 1.49%). C-peptide was assayed in duplicate 25 μL aliquots with double-antibody radioimmunoassay reagents (Diagnostic Products, Los Angeles, CA, USA). Assay sensitivity was 0.318 ng/mL; mean intra-assay CV was 3.57%; and mean inter-assay CV was 5.59%.

### 2.3. Intravenous Glucose Tolerance Test (IVGTT)

The IVGTT was conducted on an in-patient basis in the Clinical Research Unit after an overnight fast. Prior to testing, flexible intravenous catheters were placed in the antecubital spaces of both arms. Three, 2.0 mL blood samples were taken over a 20 min period for determination of basal glucose and insulin (the average of the values was used for basal “fasting” concentrations). At time 0, glucose (50% dextrose; 11.4 g/m^2^) was administered intravenously. Insulin (0.02 U/kg, Humulin; Eli Lilly, Indianapolis, IN, USA) was injected from 20 to 25 min post glucose injection. Blood samples (2.0 mL) were then collected at the following times (min) relative to glucose administration: 2, 3, 4, 5, 6, 8, 10, 12, 15, 19, 20, 21, 22, 24, 26, 28, 30, 35, 40, 45, 50, 55, 60, 70, 80, 100, 120, 140, 180. Sera were stored at −85 °C.

Glucose and insulin values were entered into the MINMOD computer program (version 3, Richard N. Bergman) for determination of the insulin S_I_ [11]. S_I_ is a composite measure of insulin-stimulated glucose uptake and insulin suppression of endogenous glucose production, and as such reflects “whole body” insulin sensitivity. It is defined as the increase of the fractional turnover of glucose per unit increase of insulin concentration. The AIRg was calculated as the incremental insulin area-under-the-curve from minutes 0 to 10 following glucose injection using the trapezoidal method. The disposition index (DI) is expressed as S_I_ X AIRg. Whole body and hepatic insulin clearance was determined using the method of Polidori et al. [12].

### 2.4. Liquid Meal Tolerance Test (MMTT)

Participants were required to fast for 12 h prior to the test, which was performed starting at between 0700 and 0800 h. To perform the test, a flexible intravenous catheter was placed in the antecubital space of one arm. At time “zero”, a liquid meal was provided (Carnation Instant Breakfast and whole milk). The meal was calculated to provide 7 kcal/kg of body weight as 24% fat, 58.6% CHO, and 17.4% protein (mean glucose ingested was 57 g; range 36–78 g). Participants were required to consume the meal within 5 min. Blood was drawn at −15 and −5 min before initiation of meal consumption (time “zero”); every 5 min from time zero to 30 min; every 10 min from 30 to 180 min; and at 210 and 240 min. Sera were stored at −85 °C. The IVGTT and MMTT were completed within a 7-day period.

### 2.5. Liquid Meal Tolerance Test Postprandial Curve Parameters

Peak (absolute maximum) and nadir (absolute minimum) values, and area-under-the-curve (AUC) of glucose and insulin were calculated from the 240 min postprandial curve reconstructed using cubic splines [13] from the sampled time points. Peak and nadir values were computed by solving for the roots of the first derivative. AUC was computed using the trapezoidal rule.

### 2.6. Reactive Hypoglycemia

RH is traditionally described as a rapid decline in blood glucose 2–5 h after food intake, often followed by counter-regulatory responses that increase hepatic glucose production [1]. However, this definition is broad and lacks physiological parameters that can be quantitatively applied. Based on prior literature and our study observations, we hypothesized that RH is characterized by a low glucose value occurring soon after the first postprandial glucose peak in combination with a steep rate of glucose decline. To translate this hypothesis into measurable criteria, we used calculus-based metrics resulting from each participant’s MMTT glucose curve to derive the following criteria for RH in our study cohort.

Using cubic spline interpolation, we identified the following for each participant: (1) the first local maximum in glucose concentration; (2) the first local minimum following that peak; and (3) the average rate of glucose decline between these two points. In our cohort of otherwise healthy adults with obesity, the median rate of decline was −1.2 mg/dL/min. Guided by this distribution and qualitative definitions in the literature emphasizing the glucose nadir [1], we defined reactive hypoglycemia using the following criteria.

Participants were classified as exhibiting RH if either of the following conditions was met:

(1) the post-peak rate of glucose decline was ≤−1.2 mg/dL per minute, and the early nadir—defined as the minimum glucose value occurring after the post-peak maximum but before 60 min—was at least 5 mg/dL below the participant’s fasting (baseline) glucose concentration;

OR

(2) the absolute minimum glucose concentration was ≤78 mg/dL and occurred within the first 80 min of observation.

Participants who did not meet either criterion were classified as not exhibiting RH.

This definition extends beyond a single nadir cut point, acknowledging that symptoms can occur both above and below glucose thresholds and that the dynamics of glucose decline are central to RH. In this population, individuals classified with RH demonstrated a faster early postprandial decline and a nadir occurring early in the postprandial period (before ~80 min), consistent with the hypothesized physiological pattern.

### 2.7. Appetite

Perceived hunger and fullness were assessed by self-report (adapted from [14,15]) during the liquid meal test. Participants responded to each of the two questions prior to the MMTT and at 15, 60, 90, 120, 180, and 240 min after the MMTT by marking a visual analog scale (VAS) ranging from zero mm (lowest) to 100 mm (highest).

### 2.8. Statistical Analysis

#### 2.8.1. Missing Time Point Concentrations in MMTT Data

Some MMTT postprandial glucose and insulin concentration values were missing at isolated time points due to various reasons such as difficulty with IV access, or sample hemolysis. We used the algorithm published in [16] through the web-based software to fill in the missing values.

#### 2.8.2. Comparison of Means Between RH and Non-RH Groups

Descriptive characteristics were done using unpaired *t*-tests for BMI and age and two-proportion z-test to evaluate differences in sex and race (proportion AA) between RH and non-RH groups.

Our primary hypothesis was that there is a difference between RH and non-RH groups in S_I_, AIRg, insulin clearance, and/or DI from the IVGTT. To test this hypothesis, we performed a two-tailed unpaired *t*-test between groups. Unpaired *t*-tests were also used to determine differences in means in the following curve parameters from the MMTT: 240 min Glucose AUC, Glucose Peak, Timing of Glucose Peak, Glucose Nadir, Timing of Glucose Nadir, 240 min Insulin AUC, Insulin Peak, Timing of Insulin Peak, Insulin Nadir, Timing of Insulin Nadir, and Initial Insulin Peak.

Assuming a moderate to large effect size (Cohen’s d = 0.50 to 0.80), α=0.05, and 80% power to obtain the range of sample size, n = 26 to n = 64 in each group was required to detect a difference a means.

#### 2.8.3. Logistic Regression Models

The dataset was imbalanced with over twice as many non-RH participants than those classified with RH. To balance the dataset for logistic regression, we randomly down-sampled the number of non-RH participants to be equal to the number of RH participants. For each category of logistic regression model, we performed the random down-sampling approach 30 times and plotted the resulting operating characteristic (ROC) curve and computed the AUC.

For each logistic regression model, we calculated model coefficients, statistical inference metrics, and the model performance metrics of McFadden’s R^2^, likelihood ratio R^2^ (R^2^L), and the Akaike Information Criterion (AIC). The mean and range of AUC values across iterations were reported for each model to capture variability in predictive performance.

To determine which curve metrics to include as covariates, we developed logistic regression models for each curve metric individually and evaluated the models using goodness-of-fit metrics. We considered race, gender, BMI, age, height, weight, 240 min AUC of glucose, 180 min AUC of glucose, DXA percent body fat, inverse insulin, HOMA IR, and IGT at 2h as independent variables of the model. We then conducted sequential regression models, adding variables with each model, and evaluating goodness of fit after each addition. The final model was based solely on the 240 min AUC of glucose. Area under the Receiver Operating Characteristics (AUC ROC), goodness-of-fit metrics, null deviance, residual deviance, Akaike Information Criterion (AIC), log-likelihood, log-likelihood null, G^2^ (Likelihood Ratio Test Statistic), McFadden R^2^, McKelvey and Zavoina R^2^ (r2ML), and Cragg and Uhler’s R^2^ (r2CU) were calculated. The McFadden’s R^2^ goodness of fit is reported here, while the remaining goodness-of-fit metrics are reported in the Supplemental Materials.

The 2-sided proportional z-tests and *t*-tests were performed in Python version 3.11 (2024), and logistic regression was performed in the statistical programming language R version 4.3.1 (2023).

## 3. Results

**Figure 1:**
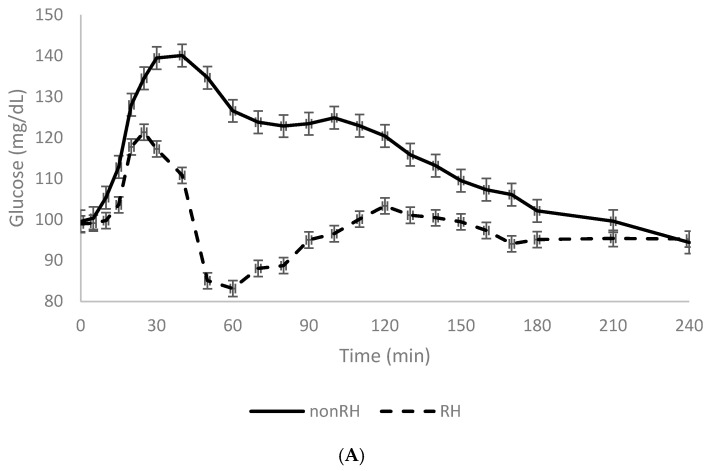

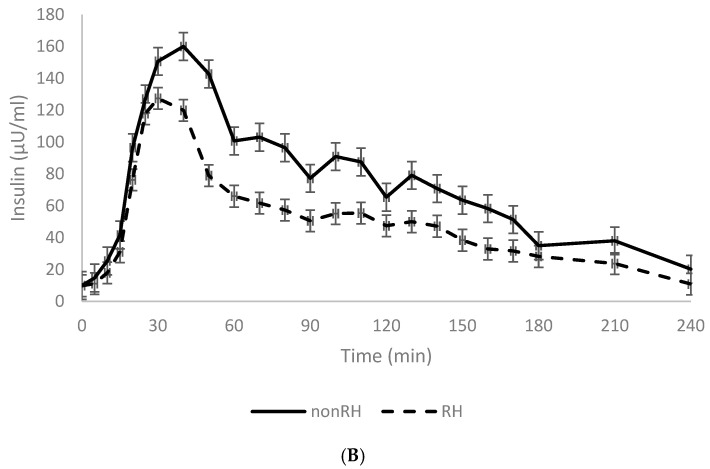
(**A**) Mean glucose and (**B**) mean insulin curves for RH and non-RH groups during the MMTT. Error bars represent the standard error of the mean (SEM).

Abbreviations and descriptions of glucose, insulin, and curve parameter variables are listed in Table 1. Baseline characteristics of the study population by non-RH (N = 51) and RH (N = 18) groups appear in Table 2. There were no significant differences in BMI, age, sex, or percent AA between the RH group and the non-RH group. Within the RH group, eleven (58%) were AA and five (26%) were male. Figure 1A shows mean glucose and Figure 1B shows mean insulin curves during the MMTT for the RH and non-RH groups.

The MMTT results were used to define RH. Curve parameter outcomes are shown in Table 3. There were significant differences between RH and non-RH participants in glucose 240 min AUC, Glucose Nadir, Glucose Nadir Time, Minimum Value After Max, Timing of Absolute Minimum, and Absolute Minimum Value.

IVGTT results were used to characterize RH and are reported in Table 4. There was no difference in AIRg between RH and non-RH participants. There were significant differences between RH and non-RH participants in S_I_ and DI. There was no significant difference in insulin clearance or in perceived hunger or fullness between RH and non-RH groups.

### Logistic Regression Models

**Figure 2:**
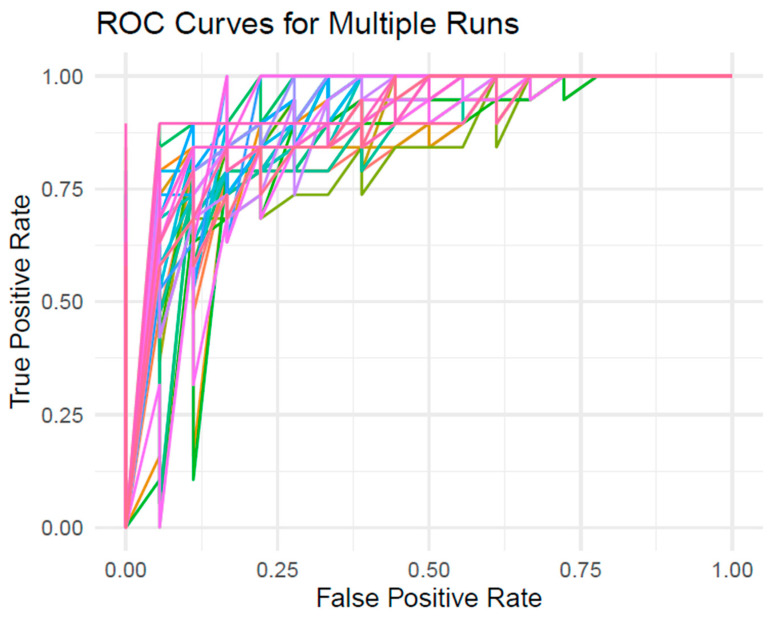
Receiver operator characteristic curves (ROC) for logistic regression models with three different sets of covariates. The three sets of covariates are Model S1: Sex, Race, Age, BMI; Model S2: Sex, Race, Age, BMI, Glucose AUC, and Model S3: Sex, Race, Age, BMI, Glucose AUC, Initial Insulin Peak. To ensure a balanced dataset of observations with and without reactive hypoglycemia, thirty runs of logistic regression models were run with 19 randomly selected observations from the participants without reactive hypoglycemia. Panel A depicts the ROC curves for Model S1, Panel B depicts the ROC curves for Model S2, and Panel C depicts the ROC curves for Model S3. Each colored curve represents the ROC curve from one resampled model iteration. The area under the curve (AUC) reflects the discriminatory performance of the model.

Results of the logistic regression analysis appear in Table 5. In Model S1, sex and race were significant predictors of RH. In Model S2, glucose AUC was a significant predictor of RH, displacing sex and race. Substitution of other curve parameters for glucose AUC produced a lower R-squared; thus, Model S2 as shown in Table 5 had the strongest predictive ability. The Receiver operator characteristic curves (ROC) for logistic regression Model S2 is shown in Figure 2. 

## 4. Discussion

The objectives of this study were to (1) define RH using the glucose and insulin responses to an MMTT and calculus-based curve fitting parameters; (2) characterize RH using S_I_, AIRg, insulin clearance, and DI from IVGTT; and (3) evaluate the postprandial hunger and fullness responses in healthy adults with obesity. We hypothesized that participants with RH would have greater S_I_, AIRg, and DI, lower clearance, and earlier return to hunger. We were able to define RH in this population using calculus-based curve parameters and found that participants with RH had higher S_I_ and DI, indicating that individuals with RH have greater sensitivity to insulin and greater insulin secretion per degree of insulin sensitivity, but did not have higher AIRg or lower insulin clearance. This study provides a unique contribution to the field that characterizes RH by curve fitting with a mixed meal. In contrast to most previous studies relying on OGTT or simple glucose nadirs, we used physiological and mathematical data to identify RH, particularly rate of glucose decline. Results suggest that RH may not just be a pathological state but may represent a distinct metabolic phenotype associated with high insulin sensitivity.

Our first objective of this study was to define RH in this dataset using curve parameters from the glucose and insulin response to the MMTT with the goal of identifying parameters that can be used to predict RH in future studies. Traditional definitions of reactive hypoglycemia (RH) describe a fall in glucose 2–5 h after eating but lack precise, physiologic parameters. Our calculus-based approach provides an objective, quantitative framework to characterize RH dynamics during the MMTT. Each participant’s glucose and insulin curves were modeled using cubic spline interpolation. Both the timing and rate of postprandial glucose change were identified. Individuals classified as RH exhibited a steeper glucose decline after the initial peak (median rate < –1.4 mg/dL/min), reaching a lower nadir that occurred earlier (<80 min) and fell ≥5 mg/dL below fasting levels compared with non-RH participants. These kinetic differences occurred despite lower overall glucose and insulin AUCs, earlier glucose and insulin peaks, and lower nadir values, indicating that RH reflects a more rapid and exaggerated transition from peak to trough glucose rather than absolute hyperinsulinemia. This calculus-based definition captures the dynamic nature of RH—integrating both the magnitude and velocity of glucose decline—and therefore more accurately identifies individuals prone to early postprandial hypoglycemia than static glucose cut points alone. Hypoglycemia is commonly diagnosed as <70 mg/dL, while more stringent cutoff values (e.g., <54 mg/dL) are considered for defining clinically significant episodes [6,10]. Our findings suggest RH can occur at higher glucose levels and that the rate of glucose decline is an important criterion in diagnosing RH. Others have reported symptoms of RH without significant glucose decline [2]. Therefore, current diagnostic criteria of RH may not be accurate for all individuals. By quantifying the rate, depth, and timing of the glucose fall, our criteria better represent the underlying physiology of RH in otherwise healthy adults with obesity and is an informative first step in mathematically predicting RH. However, our definition of RH is based on our small sample of individuals with obesity. Validation in a larger sample is needed before our MMTT-based criteria can be implemented. A future goal is to investigate RH in the National Institute of Health (NIH) Nutrition for Precision Health study population using these parameters and a much larger sample size.

In characterizing RH using IVGTT outcomes, our a priori hypothesis was that individuals with RH would have greater S_I_, AIRg, DI, and lower clearance compared to non-RH. Results indicated that the RH group had greater S_I_ and DI compared to the non-RH group. There was no difference in AIRg or clearance between groups. This suggests that high sensitivity to insulin may play a role in the pathogenesis of RH. Previous studies have reported higher insulin sensitivity in individuals with RH [2,17,18]. Using the hyperinsulinemic euglycemic clamp method, Tamburrano et al. showed elevated insulin-stimulated glucose uptake in 10 of 16 individuals with RH. They also reported increased non-oxidative glucose metabolism that explained most of the increase in glucose disposal in RH [18,19]. Greater DI among RH from the IVGTT indicates greater insulin action [20], or that the acute insulin response was not down-regulated to the extent that would be expected based on their high insulin sensitivity, resulting in a higher DI. While insulin clearance was not significantly different between the RH and non-RH groups, the RH group tended to have lower insulin clearance (*p* = 0.1), warranting further investigation. Lower insulin clearance would suggest prolonged circulating insulin action, and, when combined with greater insulin sensitivity, this extended insulin availability may exaggerate postprandial glucose decline, resulting in reactive hypoglycemia. Collectively, these findings indicate that RH is characterized not by overall insulin hypersecretion, but rather by elevated insulin sensitivity leading to postprandial glucose instability.

Logistic regression analysis was used to identify significant predictors of RH in this dataset. In Model S1, sex and race, but not age, and BMI, were significant predictors. When glucose AUC was added in Model S2, sex and race were no longer significant predictors. These results may indicate that women and AA have lower glucose AUC, suggesting that glucose AUC mediates the effects of sex and race. In this cohort, the majority of individuals in the RH group were AA women. Therefore, adding glucose AUC to Model S2 provides an explanation for why sex and race were significant in Model S1. High sensitivity to insulin may contribute to the prevalence of women in the RH group. Numerous studies have shown than women have higher insulin sensitivity than men [21]. A previous study reported greater whole body insulin sensitivity in women which was largely attributed to greater insulin-stimulated glucose uptake by skeletal muscle [22]. Additionally, AA compared to EA as a group, have higher AIRg [23], which has been attributed to both greater beta-cell responsivity and lower hepatic insulin extraction [24]. This greater AIRg is disproportionate to S_I_, thus producing a high DI. As discussed above, high DI seems to be the critical characteristic of RH associated with hypoglycemia. Thus, RH can be attributed to DI, which in turn is a product of enhanced insulin sensitivity (common among females), enhanced AIRg (common among AA), or both. Glucose AUC was a significant predictor of RH in Model S2, and was significantly lower in the RH group compared to non-RH. This finding aligns with greater insulin sensitivity and DI, and lower glucose minimum, in RH compared to non-RH. It is likely that greater insulin sensitivity and insulin action result in a more rapid decrease in glucose that causes RH.

We were also interested in looking at ratings of hunger and fullness between RH and non-RH because earlier return to hunger may lead to increased food intake during the day, which may facilitate weight gain. In this sample (both RH and non-RH combined), we previously reported earlier return to hunger after consuming a high carbohydrate, low fat meal compared to a low carbohydrate, high fat meal, and greater hunger was explained by an earlier rise and fall in postprandial glucose [25]. This suggests that the timing of the rise and fall of glucose after a meal may be particularly important to perceived hunger. A study by Bermingham et al. found bigger “dips” in glucose following meals with a higher percent energy from sugar and a lower percent energy from protein and fiber. They also found that “dips” in glucose >10% after breakfast predicted greater total daily energy intake of 166 kilocalories [26]. In this study, we did not detect greater hunger in the RH vs. non-RH group, despite the RH group having lower glucose. However, our sample size in the RH group was small, which may have limited our ability to detect a between-group difference. Further research is needed to determine if there is a relationship between RH and hunger/fullness, and whether consuming meals lower in carbohydrates and higher in fat may mitigate the decline in glucose displayed by RH.

Strengths of this study include use of MMTT for defining RH, and use of IVGTT for characterizing RH for measures of S_I_, AIRg, DI, and insulin clearance. The most common method for diagnosing RH is the oral glucose tolerance test (OGTT), however, it has been argued that the OGTT evokes a non-physiological response and may result in false-positive diagnosis of RH [9]. Brun et al. suggest that a mixed meal tolerance test (MMTT) better represents the true occurrence of RH in everyday life because it provides a macronutrient composition like that of a typical meal [9]. The strict inclusion criteria of sedentary non-diabetic overweight and obese adults limit the generalizability of these results. Future longitudinal studies including lean and active individuals are needed to fully characterize RH and determine the risk for T2D and weight gain. The MMTT was dosed according to body weight, therefore larger individuals received a bigger dose which may have affected glucose and insulin responses to the meal. RH symptoms were not recorded in the postprandial period of the MMTT; therefore, further research is required to understand the occurrence of symptoms and their temporal relationship to glucose dynamics over the meal test period.

A limitation of this study is that the number of observations in the RH group fell slightly short of the sample size designated by the power calculation (19 observations versus 25 observations). Despite this, the *p*-values indicated statistical significance, providing support for the findings. Additionally, performing multiple iterations for the logistic regression models with a balanced dataset also provides support for the average AUC values increasing as covariates associated with the MMTT were included. Taken together, these findings provide confidence that the MMTT and IVGTT provide phenotypic characteristics that differentiate between RH and non-RH populations. We did not obtain measurement of counter-regulatory hormones such as glucagon, epinephrine, cortisol, and growth hormone or reactive hypoglycemia symptom assessment, which is also limitation of this study.

## 5. Conclusions

In conclusion, we developed curve parameters to mathematically (and automatically) identify individuals with a reactive hypoglycemic response to an MMTT in our study population. Additionally, we determined that, among non-diabetic men and women with obesity, RH was characterized by greater S_I_ and higher DI from IVGTT. These findings indicate that RH is characterized not by overall insulin hypersecretion, but by greater insulin sensitivity and DI, leading to postprandial glucose instability. These findings offer a novel means to study RH as a possible early marker of a metabolic phenotype that could precede or contribute to later insulin resistance and weight gain. Our novel RH definition based on MMTT-derived curve parameters should be replicated in larger, racially and ethnically diverse cohorts to establish clinically meaningful cut points, including evaluation across sex and race, and to assess robustness and generalizability across heterogeneous metabolic phenotypes. Future research is needed to determine if the action of insulin is mechanistically involved in RH, whether RH contributes to the development of obesity and/or T2D, and to develop diet recommendations to minimize RH. 

## Figures and Tables

**Table 1:** List of abbreviations and descriptions of glucose, insulin, and curve parameter variables.

Variable	Definition of Variable	Units
Glucose peak	Absolute maximum of glucose defined as the highest observed plasma glucose concentration during the MMTT	mg/dL
Glucose peak time	Time to glucose peak defined as time from meal consumption to glucose peak	min
Glucose nadir	Absolute minimum of glucose defined as the lowest observed plasma glucose concentration during the MMTT	mg/dL
Glucose nadir time	Time to glucose nadir defined as the time from meal consumption to glucose minimum	min
Glucose AUC	Area under the curve of glucose defined as the total area under the plasma glucose concentration–time curve over the MMTT period	mg/dL·min
Initial glucose peak	First postprandial local maximum in glucose concentration	mg/dL
Initial glucose peak time	Time from meal ingestion to first glucose local maximum value	min
Initial glucose nadir	First postprandial local minimum in glucose concentration following initial glucose peak	mg/dL
Initial glucose nadir time	Time from meal ingestion to initial glucose nadir	min
Speed of Gluocse Decline	Slope of glucose decrease from initial glucose peak to initial glucose nadir (ΔG_drop ÷ Δtime)	Mg/dL·min^−1^
Glucose Min Value After Max (Before 60 min)	Early minimum before 60 min (captures rapid early dip)	mg/dL
Initial insulin peak	First postprandial local maximum in insulin concentration	µU/mL
Initial insulin peak time	Time of meal ingestion to initial inusulin peak	min
Initial insulin nadir	First postprandial local manimum in insulin concentration following initial insulin peak	µU/mL
Initial insulin nadir time	Time of meal ingestion to initial insulin nadir	min

**Table 2:** Characteristics of study population for non-reactive hypoglycemia (non-RH) and reactive hypoglycemia (RH) participants are reported as sample size, mean ± SD. Results of the two-proportion z-tests and *t*-tests are between the two groups appear in the last column.

	Non-RH	RH			
	n (%)	n (%)	Two-Proportion z-Test	95% CI	*p*
Total	50 (74%)	19 (26%)			
Race (AA) % (n)	22 (44%)	11 (58%)	−1.04	−0.38, 0.12	0.06
Sex (male)	26 (52%)	5 (26%)	−2.08	−0.47, 0.00	0.04
	**n, mean ± SD**	**n, mean ± SD**	***t*-Test**		
BMI (kg/m^2^)	50, 32.39 ± 4.01	19, 32.72 ± 4.96	0.25	−2.34, 3.00	0.802
Age (years)	50, 35.72 ± 8.42	19, 33.50 ± 7.98	1.00	−6.74, 2.31	0.325

**Table 3:** Curve parameter outcomes for RH and non-RH participants are reported as sample size, mean ± SD.

	Non-RH	RH			
	mean ± SD	mean ± SD	T-Statistic	95% CI	*p*
C-peptide 240 min AUC	1776.51 ± 614.35	1402.30 ± 555.28	2.39	−692.93, −55.48	0.023
Glucose 240 min AUC	27,340.69 ± 3147.44	23,237.99 ± 2691.64	5.31	−5671.53, −2533.85	*p* < 0.0001
Insulin 240 min AUC	17,062.99 ± 8264.52	12,353.10 ± 8270.25	2.08	−9340.70, −79.07	0.046
Glucose Peak (mg/dL)	152.62 ± 24.57	129.67 ± 25.18	3.35	−36.98, −8.93	0.002
Glucose Peak time (min)	48.78 ± 32.63	47.94 ± 37.99	0.08	−21.49, 19.81	0.934
Glucose Nadir (mg/dL)	89.61 ± 11.40	71.38 ± 10.25	6.30	−24.12, −12.34	*p* < 0.0001
Glucose Nadir time (min)	131.00 ± 77.62	72.39 ± 35.22	4.29	−85.94, −31.28	*p* < 0.0001
Insulin Peak (µU/mL)	190.40 ± 99.82	163.70 ± 99.69	0.98	−82.54, 29.15	0.337
Insulin Peak time (min)	49.39 ± 33.26	36.17 ± 18.19	2.09	−25.91, −0.54	0.041
Insulin Nadir (µU/mL)	10.99 ± 8.15	8.62 ± 5.99	1.30	−6.03, 1.30	0.200
Insulin Nadir time (min)	58.95 ± 78.75	66.72 ± 87.26	−0.33	−40.07, 55.63	0.741
Speed of Glucose Decline	−1.46 ± 2.01	−2.43 ± 2.03	−1.79	−2.44, 0.49	0.083
Glucose Min Value After Max (Before 60 min)	121.69 ± 22.03	74.74 ± 24.89	−7.22	−64.25, −29.65	*p* < 0.0001
Glucose nadir time	131.06 ± 78.78	75.32 ± 34.24	−4.09	−92.98, −18.51	*p* < 0.0001
Glucose nadir	90.42 ± 10.15	70.21 ± 10.60	−7.16	−27.80, −12.63	*p* < 0.0001

**Table 4:** IVGTT outcomes for RH and non-RH participants are reported as sample size, mean ± SD.

	n, mean ± SD	n, mean ± SD	*p* Value
Fasting Insulin	12.74 ± 7.13	10.32 ± 4.40	0.17
Fasting Glucose	98.60 ± 8.83	98.47 ± 12.08	0.96
S_I_	1.95 ± 1.44	3.25 ± 2.22	0.007
DI	47, 1490.15 ± 978.97	17, 3046.59 ± 2041.50	0.002
AIRg	48, 1039.02 ± 794.12	17, 1329.65 ± 924.81	0.72

**Table 5:** Logistic regression results evaluating the relationship between 240 min glucose area under the curve (AUC) and the likelihood of the outcome of interest, with odds ratios (OR), 95% confidence intervals, and model performance metrics reported.

	Odds Ratio	*p* > z	95% Conf. Interval	
240 min AUC Glucose	0.9995	*p* < 0.001	0.9992–0.9998	McFadden’s R^2^: 0.29 AUC ROC:0.84
Intercept	98,978.2100	*p* < 0.001	160.6011–61,000,000.0000	

## Data Availability

The data presented in this study are not publicly available due to ethical and privacy restrictions related to human participant data. De-identified data supporting the findings of this study are available from the corresponding author upon reasonable request and with appropriate institutional approvals.

## Supplementary Materials

The following supporting information can be downloaded at: https://www.mdpi.com/article/10.3390/nu18050822/s1, Model S1: Covariates: Sex, Race, Age, BMI; Model S2: Covariates: Sex, Race, Age, BMI, Glucose AUC; Model S3: Covariates: Sex, Race, Age, BMI, Glucose AUC, Glucose Peak.
