# Supplementary material for: Defining and Characterizing Postprandial Reactive Hypoglycemia
Source: Nutrients. 2026 Mar 3;18(5):822. doi: 10.3390/nu18050822 (PMC12986748; doi:10.3390/nu18050822)
Supplement: Supplementary file 1 [file nutrients-18-00822-s001.zip › nutrients-4127908-supplementary.pdf]

Supplementary Materials: Full Summary of Logistic Regression Models

Model S1:
Covariates: Sex, Race, Age, BMI

Model with maximum AUC value

Covariates	Estimate	Std. Error	z value	Pr(>|z|)
(Intercept)	-0.0821	3.3155	-0.0248	0.9803
Sex	-1.6905	0.8067	-2.0956	0.0361
Race	1.9354	0.9757	1.9837	0.0473
Age	0.0638	0.0504	1.2662	0.2055
BMI	-0.0679	0.0967	-0.7029	0.4821

Dispersion parameter for binomial family taken to be 1
Null deviance: 51.2659 on 36 degrees of freedom
Residual deviance: 41.3747 on 32 degrees of freedom
AIC: 51.3747
Number of Fisher Scoring iterations: 4
Log-likelihood: -20.6873
Log-likelihood Null: -25.6329
G2: 9.8912
McFadden R2: 0.1929
r2ML: 0.2346
r2CU: 0.3128

Model with minimum AUC value

Covariates	Estimate	Std. Error	z value	Pr(>|z|)
(Intercept)	1.1773	3.1177	0.3776	0.7057
Sex	-0.6597	0.7441	-0.8866	0.3753
Race	0.3288	0.702	0.4683	0.6396
Age	-0.0237	0.0443	-0.5349	0.5927
BMI	-0.0087	0.0781	-0.1116	0.9111

Dispersion parameter for binomial family taken to be 1
Null deviance: 51.2659 on 36 degrees of freedom
Residual deviance: 49.989 on 32 degrees of freedom
AIC: 59.989
Number of Fisher Scoring iterations: 4
Log-likelihood: -24.9945
Log-likelihood Null: -25.6329

G2: 1.2768
McFadden R2: 0.0249
r2ML: 0.0339
r2CU: 0.0452

Model S2:
Covariates: Sex, Race, Age, BMI, Glucose AUC

Model with maximum AUC value

Covariates	Estimate	Std. Error	z value	Pr(>|z|)
(Intercept)	19.8665	9.9782	1.991	0.0465
Sex	-1.8873	1.5866	-1.1895	0.2342
Race	2.8519	1.5127	1.8853	0.0594
Age	0.1396	0.0849	1.6444	0.1001
BMI	0.091	0.1962	0.464	0.6427
Glucose AUC	-0.0011	0.0004	-2.4036	0.0162

Dispersion parameter for binomial family taken to be 1
Null deviance: 51.2659 on 36 degrees of freedom
Residual deviance: 15.3166 on 31 degrees of freedom
AIC: 27.3166
Number of Fisher Scoring iterations: 7
Log-likelihood: -7.6583
Log-likelihood Null: -25.6329
G2: 35.9492
McFadden R2: 0.7012
r2ML: 0.6215
r2CU: 0.8289

Model with minimum AUC value

Covariates	Estimate	Std. Error	z value	Pr(>|z|)
(Intercept)	7.2723	4.186	1.7373	0.0823
Sex	-0.4716	0.8751	-0.5389	0.59
Race	0.7177	0.8248	0.8702	0.3842
Age	0.0077	0.0542	0.1412	0.8877
BMI	0.0542	0.0944	0.5739	0.566
Glucose AUC	-0.0004	0.0002	-2.248	0.0246

Dispersion parameter for binomial family taken to be 1
Null deviance: 51.2659 on 36 degrees of freedom
Residual deviance: 38.7493 on 31 degrees of freedom
AIC: 50.7493
Number of Fisher Scoring iterations: 5
Log-likelihood: -19.3746

Log-likelihood Null: -25.6329
G2: 12.5166
McFadden R2: 0.2442
r2ML: 0.287
r2CU: 0.3828

Model S3:
Covariates: Sex, Race, Age, BMI, Glucose AUC, Glucose Peak
<u>Model with maximum AUC value</u>

**Covariates**	**Estimate**	**Std. Error**	**z value**	**Pr(>|z|)**
(Intercept)	40.8151	23.4747	1.7387	0.0821
Sex	-4.7348	2.5324	-1.8697	0.0615
Race	5.5165	3.4869	1.5821	0.1136
Age	-0.0906	0.1123	-0.8069	0.4197
BMI	-0.1785	0.3547	-0.5033	0.6147
Glucose AUC	-0.0021	0.0012	-1.8283	0.0675
Glucose Peak	0.1647	0.107	1.5394	0.1237

Dispersion parameter for binomial family taken to be 1
Null deviance: 51.2659 on 36 degrees of freedom
Residual deviance: 15.9611 on 30 degrees of freedom
AIC: 29.9611
Number of Fisher Scoring iterations: 8
Log-likelihood: -7.9805
Log-likelihood Null: -25.6329
G2: 35.3048
McFadden R2: 0.6887
r2ML: 0.6149
r2CU: 0.82
Null deviance: 52.679 on 37 degrees of freedom
Residual deviance: 18.950 on 31 degrees of freedom
AIC: 32.95
Number of Fisher Scoring iterations: 7

<u>Model with minimum AUC Value</u>

**Covariates**	**Estimate**	**Std. Error**	**z value**	**Pr(>|z|)**
(Intercept)	8.2572	5.112	1.6153	0.1063
Sex	-0.2532	0.8978	-0.2821	0.7779
Race	-0.0393	0.9906	-0.0396	0.9684
Age	0.08	0.0737	1.0859	0.2775
BMI	0.0258	0.1188	0.2168	0.8283
Glucose AUC	-0.0005	0.0003	-1.9976	0.0458

Glucose Peak	0.0128	0.0422	0.3043	0.7609

Dispersion parameter for binomial family taken to be 1
Null deviance: 51.2659 on 36 degrees of freedom
Residual deviance: 38.1259 on 30 degrees of freedom
AIC: 52.1259
Number of Fisher Scoring iterations: 4
Log-likelihood: -19.063
Log-likelihood Null: -25.6329
G2: 13.1399
McFadden R2: 0.2563
r2ML: 0.2989
r2CU: 0.3987
